# Resolving the dilution paradox to improve the interpretation of extracellular vesicle biomarker studies

**DOI:** 10.1016/j.rpth.2026.106636

**Published:** 2026-05-08

**Authors:** Joyce Rops, Naomi C. Buntsma, Aleksandra Gąsecka, Rienk Nieuwland, Aleksandra Rosiek, Yvo B.W.E.M. Roos, A. Yaël Nossent, Nyika D. Kruyt, Edwin van der Pol, Ceren Eyileten

**Affiliations:** 1 Laboratory of Experimental Clinical Chemistry, Laboratory Specialized Diagnostics & Research, Department of Laboratory Medicine, Amsterdam UMC, University of Amsterdam, Amsterdam, Netherlands; 2 Biomedical Engineering & Physics, Amsterdam UMC, University of Amsterdam, Amsterdam, Netherlands; 3 Amsterdam Cardiovascular Sciences, Atherosclerosis and Ischemic Syndromes, Amsterdam, Netherlands; 4 Department of Neurology, Leiden University Medical Center, Leiden, Netherlands; 5 Department of Neurology, Amsterdam UMC, University of Amsterdam, Amsterdam, Netherlands; 6 Amsterdam Neuroscience, Neurovascular Disorders, Amsterdam, Netherlands; 7 Department of Cardiology, Medical University of Warsaw, Warsaw, Poland; 8 Amsterdam Cardiovascular Sciences, Microcirculation, Amsterdam, Netherlands; 9 Cancer Center Amsterdam, Imaging and Biomarkers, Amsterdam, Netherlands; 10 Department of Child and Adolescent Psychiatry, Institute of Psychiatry and Neurology, Warsaw, Poland; 11 Amsterdam Neuroscience, Neurovascular Disorders, Amsterdam, Netherlands; 12 Department of Nutrition, Exercise and Sports, University of Copenhagen, Copenhagen, Denmark; 13 Department of Experimental and Clinical Pharmacology, Centre for Preclinical Research and Technology (CePT), Medical University of Warsaw, Warsaw, Poland; 14 Genomics Core Facility, Center of New Technologies (CeNT), University of Warsaw, Warsaw, Poland

**Keywords:** biomarkers, extracellular vesicles, flow cytometry, myocardial infarction, stroke

## Abstract

**Background:**

Concentrations of extracellular vesicles (EVs) and other particles are measured in plasma for biomarker exploration. A commonly used method, flow cytometry, requires plasma dilution to ensure single-particle detection. Since plasma EVs are outnumbered by variable concentrations of lipoproteins, dilution differs between the plasma samples. Dilution can result in misidentification of fluorescent background signals as labeled EVs. This phenomenon, called the dilution paradox, leads to overestimation of plasma EV concentrations, and likely impacts conclusions from earlier performed biomarker studies.

**Objectives:**

This study reevaluated earlier conclusions from our clinical biomarker studies Antiplatelet Therapy Effect on Extracellular Vesicles (AFFECT EV) and Circulating Nanotraces to Identify the Cause of Stroke (CINTICS), by taking the dilution paradox into account.

**Methods:**

We developed a model that quantifies the fluorescent background and estimates whether a flow cytometry measurement is dominated by fluorescent background, that is, if the measurement is unreliable. This model was applied to the original datasets of the AFFECT EV and CINTICS studies to identify and exclude unreliable measurements. We investigated whether exclusion of unreliable data affects the original conclusions.

**Results:**

Our model estimated that 47% (1156/2457) of the evaluated measurements are unreliable, and conclusions from both biomarker studies required adjustment.

**Conclusion:**

Our model improves reliability and reproducibility of EV concentration measurements using flow cytometry. We recommend to reanalyze earlier EV flow cytometry studies using our model and to use a fixed dilution factor in future EV flow cytometry studies to enable reliable EV concentration measurements.

## Essentials

• Extracellular vesicle (EV) detection by flow cytometry can be unreliable due to plasma dilution.
• A model was developed to identify and exclude unreliable measurements of EVs.
• Conclusions from published biomarker studies changed after applying our model.
• Our model improves reliability, comparability, and reproducibility of measured EV concentrations.

## 1 Introduction

Extracellular vesicles (EVs) are membrane-delimited particles that are naturally released by various cell types, such as platelets and leukocytes. In recent years, EVs have gained interest as potential biomarkers in various diseases, because their size, composition, and release rate can change under pathological circumstances [1]. However, the accurate measurement of EV concentrations in body fluids such as blood plasma is challenging, due to the inherent heterogeneity of EVs in size and composition.

With flow cytometry, single particles such as EVs can be characterized directly in diluted plasma [2]. However, the detected particle concentration in plasma can differ up to 100-fold between donors [3]. To accommodate for these differences, a custom dilution factor is applied to each sample. This custom dilution factor ensures single-particle detection based on light scattering signals [4]. Concurrently, dilution can decrease the concentration of rare fluorescently labeled EV populations to below measurable levels. This contradiction creates the dilution paradox, where the required dilution factor for single-particle detection based on light scattering signals precludes the measurement of labeled EVs [5]. Consequently, EV concentration measurements are biased in samples to which the dilution paradox applies. This finding raises concerns about the reliability of data in previously published studies in which custom dilution factors were applied.

One such study is the Antiplatelet Therapy Effect on Extracellular Vesicles (AFFECT EV) study, which we published previously [6]. During acute myocardial infarction (AMI), activated blood and endothelial cells release EVs, which are procoagulant and proinflammatory [7]. These EVs contribute to clot formation and chronic inflammation. Standard post-AMI pharmacotherapy includes antiplatelet therapy, including P2Y12 inhibitors such as ticagrelor or clopidogrel. We investigated whether ticagrelor inhibits the release of procoagulant EVs more effectively than clopidogrel. Therefore, we used flow cytometry to measure EVs in blood plasma samples from patients with AMI randomized to treatment with either ticagrelor or clopidogrel.

Alongside the AFFECT EV study, the dilution paradox may also have influenced a recently published study from the Circulating Nanotraces to Identify the Cause of Stroke (CINTICS) project [8]. CINTICS is an observational study aimed to identify circulating biomarkers, such as small RNAs or EVs, to distinguish between stroke subtypes [9]. EVs may play a key role in stroke by mediating intercellular communication and promoting coagulation, inflammation, and vascular injury, thereby influencing both stroke onset and recovery processes [10,11]. Therefore, EV concentrations in peripheral blood may provide valuable information that could expedite the triage of patients with suspected stroke. Our previously published study investigated whether patients with confirmed ischemic stroke could be distinguished from those with suspected stroke with a confirmed different diagnosis, based on EV concentration measurements in plasma.

In this study, we aimed to reevaluate the AFFECT EV and CINTICS studies using a newly developed analytical model that enables the identification of flow cytometry measurements that are biased as a result of the dilution paradox. Specifically, we first used the new model to select the reliable EV concentration measurements from the data of both studies. Using these reliable measurements, we reevaluated the previous conclusions on the diagnostic utility of EVs in (i) the effects of P2Y12 inhibitors in patients after AMI and (ii) stroke subtype identification.

## 2 Methods

### 2.1 Study designs

#### 2.1.1 AFFECT EV

Details of the AFFECT EV study have been published previously [6]. In short, AFFECT EV was a single-center randomized controlled trial comparing the effect of 2 P2Y12 antagonists, ticagrelor and clopidogrel, on EV release. Blood was collected from 60 patients admitted for first ST-segment elevation of AMI or non–segment elevation of AMI with symptom onset within the previous 24 hours. Blood was collected at 3 time points as follows: (i) 24 hours after percutaneous coronary intervention (PCI), (ii) 72 hours after PCI, and (ii) 6 months after PCI. The primary endpoint was the concentration of EVs from activated platelets, and the secondary endpoints were concentrations of platelet EVs exposing fibrinogen, leukocyte-derived EVs, and endothelial cell-derived EVs at 72 hours and 6 months.

#### 2.1.2 CINTICS

All details and results of the CINTICS study have been published previously [8]. In short, CINTICS is an observational study aiming to find biomarkers, including short noncoding RNA molecules and EVs, to identify stroke and stroke subtypes. Blood was collected from 482 patients presenting to the emergency room of the Amsterdam University Medical Center, location Academic Medical Center, with a suspicion of stroke. Of these, 155 blood samples were selected for preliminary analysis. The aim of the study was to identify patients with ischemic stroke from those with suspected stroke based on circulating EV concentrations.

### 2.2 Flow cytometry measurements

#### 2.2.1 AFFECT EV

Details about sample collection and handling, and data calibration and processing, were previously published [6]. Platelet-poor plasma was measured using an Apogee A60-Micro (Apogee Flow Systems). Data represent the number of events that exceeded the side-scatter trigger threshold corresponding to a side-scattering cross-section of 10 nm^2^, have a diameter of >200 nm, have a refractive index of <1.42, and whose fluorescence signal exceeds the set gate for the corresponding marker, per milliliter of platelet-poor plasma. In this study, we included flow cytometry measurements of activated platelet-derived EVs (CD61^+^CD62p^+^), fibrinogen-exposing EVs (Fib^+^), leukocyte-derived EVs (CD45^+^), endothelial cell-derived EVs (CD31^+^CD146^+^), erythrocyte-derived EVs (CD235a^+^), and phosphatidylserine (PS)-exposing EVs.

#### 2.2.2 CINTICS

Details about sample collection and handling, and data calibration and processing, were previously published [8]. Platelet-poor plasma was measured using Cytek Northern Lights (Cytek Biosciences). Data represent the number of events that exceeded the side-scatter trigger threshold corresponding to a side-scattering cross-section of 2 nm^2^, have a diameter between 100 and 1000 nm, and whose fluorescence signal exceeds the set gate for the corresponding marker, per milliliter of platelet-poor plasma. In this study, we included flow cytometry measurements of platelet-derived EVs (CD41^+^), activated platelet-derived EVs (CD62p^+^), erythrocyte-derived EVs (CD235a^+^), leukocyte-derived EVs (CD45^+^), monocyte- and macrophage-derived EVs (CD14^+^), platelet endothelial cell adhesion molecule (PECAM)-exposing EVs (CD31^+^), epithelial cell adhesion molecule–exposing EVs (CD326^+^), and EVs exposing 4-(N-[S-glutathionylacetyl]amino)phenylarsonous acid, which is an indicator of platelet cell death.

### 2.3 The dilution paradox model

For the complete model derivation and application, please refer to [5]. In short, the model describes the relationship between the number of fluorescent particles measured by the flow cytometer and the dilution factor, that is, the extent to which a sample is diluted prior to measurement. In a typical study, it is expected that the number of fluorescent particles decreases with increasing dilution factor until it reaches the fluorescent background level, where it will remain constant. The reported EV concentrations are the result of dividing the number of fluorescent particles by the measured sample volume and multiplying the result by the dilution factor. Therefore, the presence of a constant level of fluorescent background events causes incremental overestimation of the EV concentration with the increasing dilution factor. Our analytical model determines the magnitude of the fluorescent background. Based on this fluorescent background level, the model then estimates which samples may contain fluorescent background only. These are the samples that are affected most by the dilution paradox and will be excluded from reanalysis.

### 2.4 Statistical analysis

#### 2.4.1 AFFECT EV

Included data were analyzed in the same way as published previously [6].

#### 2.4.2 CINTICS

Included data for the identification of ischemic stroke (large vessel occlusion, nonlarge vessel occlusion ischemic stroke and/or transient ischemic attacks) were statistically analyzed in the same way as published previously [8]. The only exception is the comparison of categorical variables, which were compared using Fisher exact test because of its robustness to small sample sizes compared with the originally used chi-squared test. Analysis for the identification of hemorrhagic stroke (intracerebral and/or subarachnoid hemorrhage) vs patients with confirmed different diagnoses was performed in the same way as the analysis for the identification of ischemic stroke.

### 2.5 Ethics statement

#### 2.5.1 AFFECT EV

The study protocol, designed in compliance with the Declaration of Helsinki, was approved by the Ethics Committee of Medical University of Warsaw (approval number: KB/112/2016), registered in the ClinicalTrials database (NCT02931045). All patients provided written informed consent.

#### 2.5.2 CINTICS

The study was designed in compliance with the Declaration of Helsinki and approved by the Ethical Review Board of Amsterdam UMC, location AMC, in combination with a deferred consent process (approval number NL72929.018.20). Patients were included from October 2020 to November 2022.

## 3 Results

### 3.1 Reanalysis of AFFECT EV

To select the reliably measured samples, the dilution paradox model was applied to data from the AFFECT EV study [6]. In this analysis, a measurement is considered reliable when the model estimated that the measurement did not contain fluorescent background only. Supplementary Figure 1 shows that the reliability of the measurements is different for each EV type. For example, no reliable measurements were observed of fibrinogen^+^ EVs (Supplementary Figure 1B), while all measurements of activated platelet-derived EVs (CD61^+^CD62p^+^) were reliable (Supplementary Figure 1A). All data identified as unreliable were excluded prior to reanalysis, resulting in complete exclusion of fibrinogen^+^ EV measurements. As all measurements of activated platelet-derived EVs were reliable, the dataset and conclusions remain unchanged, and no further reanalysis is needed.

**Figure 1:**
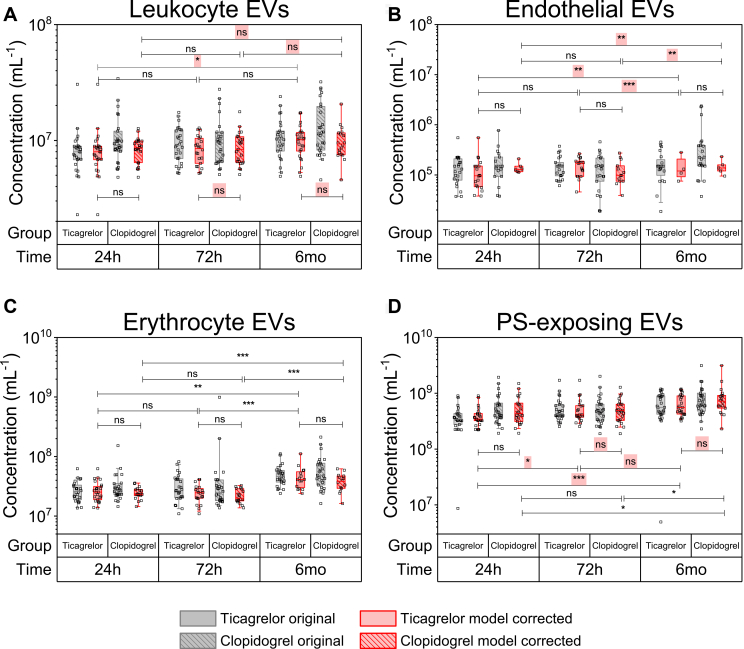
Extracellular vesicle (EV) concentrations in blood plasma of patients using ticagrelor or clopidogrel at 24 hours, 72 hours, and 6 months after onset of acute myocardial infarction. Data represents particles that (1) exceeded the trigger threshold corresponding to a side-scattering cross-section of 10 nm^2^, (2) have a diameter > 200 nm, (3) have a refractive index of < 1.42, and (4) exceed the fluorescent threshold corresponding to the used label, per milliliter. Gray boxes reflect the data as represented in the original article of Antiplatelet Therapy Effect on Extracellular Vesicles (AFFECT EV) [6], and red boxes reflect the data after application of the model. Differences that have changed from nonsignificant to significant or vice versa are highlighted. Please note that not all axis are on the same scale. Panels reflect the following EV subtypes: (A) Leukocyte-derived EVs (CD45^+^), (B) endothelial EVs (CD31^+^CD146^+^), (C) erythrocyte-derived EVs (CD235a^+^), and (D) PS-exposing EVs (lactadherin^+^). ∗*P* < .05; ∗∗*P* < .01; ∗∗∗*P* < .001. PS, phosphatidylserine.

Figure 1A shows that the concentration of leukocyte-derived (CD45^+^) EVs has increased in plasma of patients using ticagrelor at 6 months after AMI, a finding that was not found in the original publication of AFFECT EV. In addition, the originally observed differences in leukocyte-derived (CD45^+^) EV concentrations between the treatment arms are no longer significant.

Figure 1B shows an increase of endothelial EV (CD31^+^CD146^+^) concentrations in both treatment arms that was not observed in the original AFFECT EV article. No significant differences are observed between the treatment arms, which is in agreement with the original AFFECT EV article.

Figure 1C shows that the erythrocyte-derived (CD235a^+^) EV concentration increased over time in both patient groups. No statistically significant differences were observed between patients on ticagrelor and those on clopidogrel. Both of these observations are consistent with the original AFFECT EV article.

Figure 1D shows that, in both patient groups, the concentration of PS-exposing EVs increases over time, while in the original article, this increase was only visible in patients treated with clopidogrel. In contrast to the original AFFECT EV article, the concentration of PS-exposing EVs was comparable between treatment arms at all time points.

The results of our reanalysis reveal that a difference between patients treated with ticagrelor and those with clopidogrel could only be observed in the measurements of activated platelet-derived EVs (CD61^+^CD62p^+^). This refines the main finding of the AFFECT EV article, which originally stated that ticagrelor attenuates the increase of activated platelet-derived EV (CD61^+^CD62p^+^), fibrinogen^+^ EV, erythrocyte-derived (CD235a^+^) EV, and PS-exposing EV concentrations in plasma.

### 3.2 Reanalysis of CINTICS

Supplementary Figure 2 shows the application of the dilution paradox model to flow cytometry data from CINTICS. Reliability of the measurements is dependent on EV type, similar to what was observed in the reanalysis of the AFFECT EV study. For example, for PECAM-exposing (CD31^+^) EVs, almost all measurements (96%) are reliable, while for endothelial (CD146^+^) EVs, none of the measurements (0%) is reliable. Only reliable data are included for reanalysis.

#### 3.2.1 Identification of ischemic stroke

**Figure 2:**
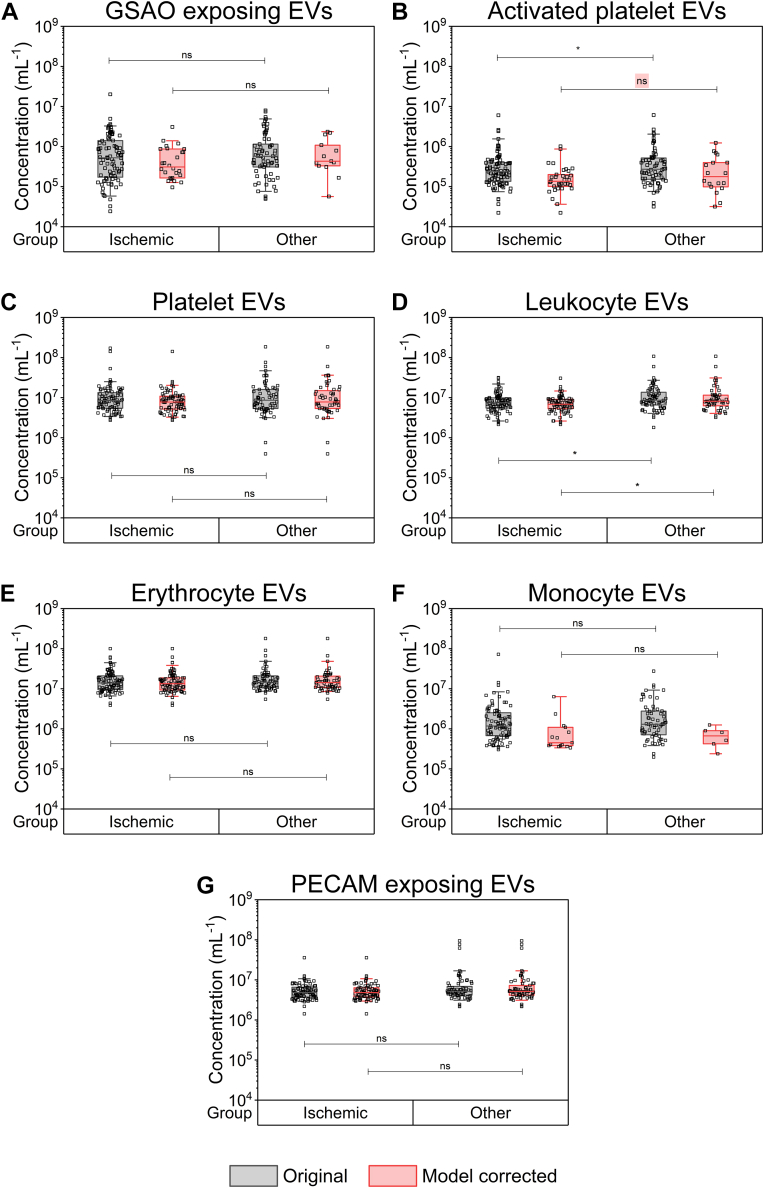
Concentrations of extracellular vesicles (EVs) in plasma of patients with and without ischemic stroke. Concentrations indicate the number of measured events that (1) exceed the trigger threshold corresponding to a side-scatter cross-section of 2 nm^2^, (2) have a diameter between 100 and 1000 nm, and (3) exceed the fluorescent threshold corresponding to the used label, per milliliter. Gray boxes reflect data as presented in the original Circulating Nanotraces to Identify the Cause of Stroke (CINTICS) article [8], and red boxes reflect data after application of the model. Differences that have changed in significance are highlighted. Panels reflect concentrations of the following EV subtypes: (A) EVs exposing cell death marker (GSAO^+^); (B) activated platelet–derived EVs (CD62p^+^); (C) platelet-derived EVs (CD41^+^); (D) leukocyte-derived EVs (CD45^+^); (E) erythrocyte-derived EVs (CD235a^+^); (F) monocytes- and/or macrophages-derived EVs (CD14^+^); (G) PECAM-exposing EVs (CD31^+^). ∗*P* < .05; ∗∗*P* < .01; ∗∗∗*P* < .001. GSAO, 4-(N-[S-glutathionylacetyl]amino)phenylarsonous acid; PECAM, platelet endothelial cell adhesion molecule.

In the original CINTICS article, leukocyte-derived (CD45^+^) EVs and activated platelet-derived (CD62p^+^) EVs were found to be significantly decreased in plasma of patients with confirmed ischemic stroke, compared with patients with confirmed different diagnoses [8]. Figure 2 shows that after application of the dilution paradox model, this decrease in leukocyte-derived (CD45^+^) EV concentration is retained (Figure 2D) (*P* = .015), while the decrease in activated platelet-derived (CD62p^+^) EV concentration is no longer significant (Figure 2B) (*P* = .295) [8]. Plasma concentrations of all other included EV types did not differ between patients with and those without ischemic stroke, which is in agreement with the previous publication. Therefore, data were excluded based solely on leukocyte-derived (CD45^+^) EV concentrations, leading to the exclusion of 40 patients (19% of data points) (Figure 2D).

The characteristics of included patients are shown in Supplementary Table 1. No differences regarding baseline characteristics, comorbidities, or laboratory data were observed between patients with ischemic stroke vs those without ischemic stroke. However, since age and diastolic blood pressure (DBP) have been used in multivariate analysis in the article of Buntsma et al. [8] and show borderline significance in univariate analysis in Supplementary Table 1 (*P* = .06), we also included age and DBP in the current multivariate analysis.

Supplementary Table 4 shows the univariate receiver-operating characteristic (ROC) analysis for the identification of ischemic stroke, which was used to determine the optimal cutoff values for age, DBP, and leukocyte-derived (CD45^+^) EV concentration. These factors were incorporated in the multivariate logistic regression analysis with their respective optimal cutoffs.

The results of multivariate analysis are shown in Table 1. In multivariate logistic regression, age ≥ 58 years was independently associated with higher odds ratio (OR) of ischemic stroke (OR, 3.32; 95% CI, 1.22-10.15; *P* = .024). DBP of ≥81 mm Hg was also an independent predictor of ischemic stroke (OR, 2.95; 95% CI, 1.29-7.07; *P* = .012). Finally, a plasma leukocyte-derived (CD45^+^) EV concentration of ≤ 7.74 × 10^6^/mL was independently associated with increased OR of ischemic stroke (OR, 2.55; 95% CI, 1.13-5.99; *P* = .027). These results are different from the results presented in the original publication of CINTICS, where the occurrence of ischemic stroke was associated with age > 70 years, DBP > 82 mm Hg and a plasma leukocyte-derived (CD45^+^) EV concentration ≤ 2.16 × 10^5^/mL [8].

**Table 1:** Results of multivariate logistic regression analysis for ischemic stroke classification using age, DBP, and leukocyte-derived EV concentrations, based on cutoff values determined with receiver-operating characteristics analysis.

Characteristic	OR (95% CI)	*P*
Age ≥ 58 y	3.32 (1.22-10.15)	**.024**
DBP ≥ 81 mm Hg	2.95 (1.29-7.07)	**.012**
CD45^+^ EV concentration ≤ 7.74 × 10^6^/mL	2.55 (1.13-5.99)	**.027**

*P* values shown in bold are statistically significant (*P* < .05).

CD, cluster of differentiation; CI, confidence interval; DBP, diastolic blood pressure; EV, extracellular vesicle; OR, odds ratio.

**Figure 3:**
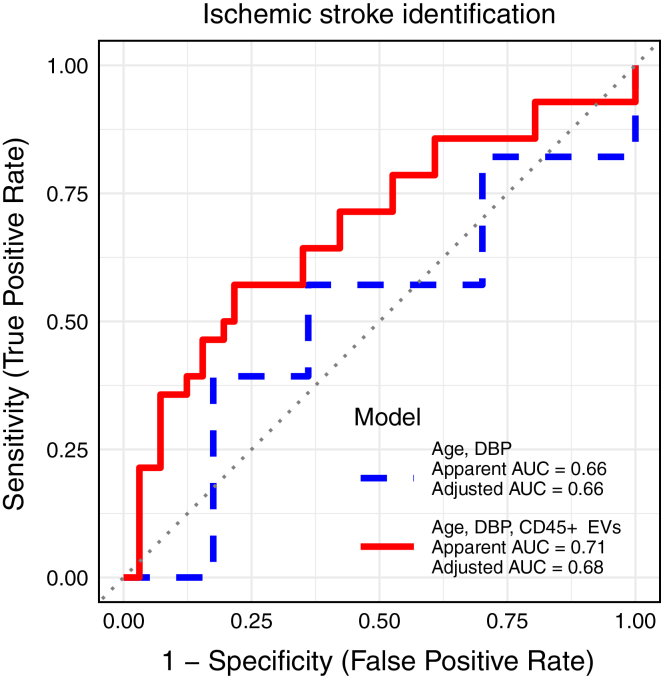
Receiver-operating characteristic (ROC) curves to distinguish between patients with confirmed ischemic stroke and patients with a confirmed different diagnosis. ROC analysis based on age and diastolic blood pressure (DBP) alone (blue dashed line) showed an apparent area under the curve (AUC) of 0.66, which remained 0.66 (95% CI, 0.58-0.74; *P* = .01) after optimism correction using bootstrap validation with 200 resamples. ROC analysis based on age and DBP in combination with the concentration of leukocyte-derived (CD45^+^) extracellular vesicles (EVs; red solid line) showed an apparent AUC of 0.71 and an optimism-adjusted AUC of 0.68 (95% CI 0.59-0.78; *P* = .01).

The added diagnostic value of leukocyte-derived (CD45^+^) EV concentrations was evaluated by comparing ROC analyses of multiple logistic regression models, including (i) only the baseline parameters age and DBP, vs (ii) baseline parameters (age and DBP) combined with leukocyte-derived (CD45^+^) EV concentrations in plasma. The results are shown in Figure 3. Adding the leukocyte-derived (CD45^+^) EV concentration to the logistic regression model significantly improved the identification of ischemic stroke. The area under the curve (AUC) increased from 0.66 to 0.70 (*P* = .027, DeLong test), with optimism-adjusted AUCs of 0.66 (95% CI, 0.58-0.74; *P* = .01) and 0.68 (95% CI, 0.59-0.78; *P* = .01), respectively. This AUC increase of 0.04 is smaller than the 0.07 AUC increase that was found in the original CINTICS publication [8].

#### 3.2.2 Identification of hemorrhagic stroke

Patient characteristics with respect to hemorrhagic stroke identification are summarized in Supplementary Table 2. Compared with patients with alternative confirmed diagnoses, patients with confirmed hemorrhagic stroke had higher leukocyte counts (*P* < .001) and higher glucose levels (*P* = .02). Additionally, 7-day mortality after emergency room admission was higher in the hemorrhagic stroke group (*P* < .001). No other differences were observed in baseline characteristics, comorbidities, pharmacotherapy, laboratory data, or outcomes between the 2 groups.

**Figure 4:**
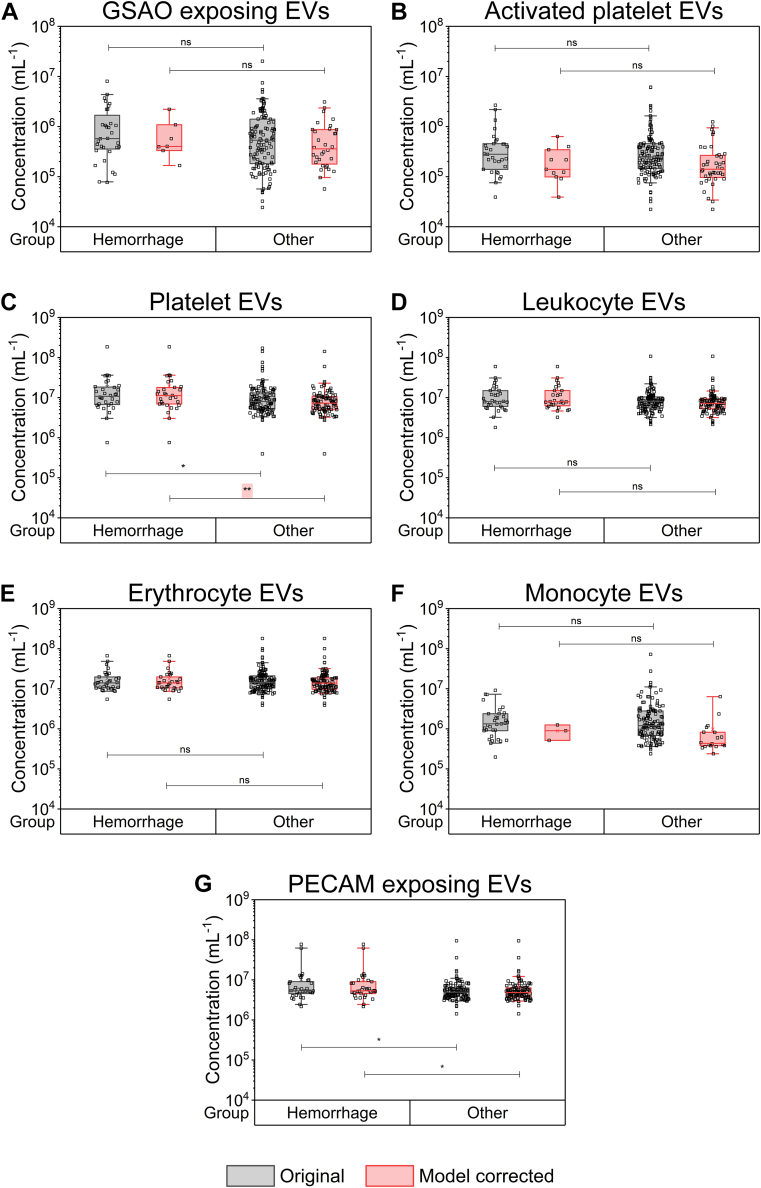
Concentrations of extracellular vesicles (EVs) in plasma of patients with or without hemorrhagic stroke. Concentrations indicate the number of measured events that (1) exceed the trigger threshold corresponding to a side-scatter cross-section of 2 nm^2^, (2) have a diameter between 100 and 1000 nm, and (3) exceed the fluorescent threshold corresponding to the used label, per milliliter. Gray boxes reflect the original dataset, and red boxes reflect data after application of the model. Differences that have changed in significance have been highlighted. Panels reflect concentrations of the following EV subtypes: (A) EVs exposing cell death marker GSAO^+^; (B) activated platelet–derived EVs (CD62p^+^); (C) platelet-derived EVs (CD41^+^); (D) leukocyte-derived EVs (CD45^+^); (E) erythrocyte-derived EVs (CD235a^+^); (F) monocytes- and/or macrophages-derived EVs (CD14^+^); (G) PECAM-exposing EVs (CD31^+^). ∗*P* < .05; ∗∗*P* < .01; ∗∗∗*P* < .001. GSAO, 4-(N-[S-glutathionylacetyl]amino)phenylarsonous acid; PECAM, platelet endothelial cell adhesion molecule.

Figure 4 shows plasma concentrations of different EV types in patients with confirmed hemorrhagic stroke vs those with other diagnoses. Compared with other patients, patients with hemorrhagic stroke have a higher plasma concentration of platelet-derived (CD41^+^) EVs (Figure 4C) (*P* = .006) and PECAM-exposing (CD31^+^) EVs (Figure 4G) (*P* = .036). The model corrected data reflect a stronger association than analysis of the original dataset of platelet-derived (CD41^+^) EVs (*P* = .035) and PECAM-exposing (CD31^+^) EVs (*P* = .042) [8]. No difference was observed in the plasma concentrations of all other EV types in patients with vs without hemorrhagic stroke. Concentrations of platelet-derived (CD41^+^) EVs and PECAM-exposing (CD31^+^) EVs were included in multivariate logistic regression analysis. Patients were excluded if no reliable measurement was available of the platelet-derived (CD41^+^) or PECAM-exposing (CD31^+^) EV concentration. Data from 121 patients (26 with hemorrhagic stroke and 95 without hemorrhagic stroke) were included in further analysis.

Baseline characteristics of included patients can be found in Supplementary Table 3. The primary goal of CINTICS is to identify biomarkers that can be measured in emergency settings, that is, at home or in ambulances. Because leukocyte count is unsuitable for such settings, it was not included in multivariate analysis despite its statistical significance (*P* < .001). Glucose levels were not included either, as it is unclear whether the observed differences (*P* = .02) are attributable to undiagnosed diabetes mellitus rather than hemorrhagic stroke itself.

Age and sex, although not statistically significant in univariate analysis (*P* = .15 and *P* = .27, respectively), were included in the multivariate analysis because they are established risk factors for hemorrhagic stroke and are readily available in emergency settings. Supplementary Table 4 presents the results of univariate ROC analysis for age, sex, PECAM-exposing (CD31^+^) EV concentration, and platelet-derived (CD41^+^) EV concentration to determine their optimal cutoff values in the identification of hemorrhagic stroke. These variables were included in multivariate analysis with their respective cutoff values.

Table 2 shows that plasma concentrations of platelet-derived (CD41^+^) EVs of ≥1.13 × 10^7^/mL are associated with increased OR of hemorrhagic stroke (OR, 2.83; 95% CI, 1.00-8.09; *P* = .049). The variables age, sex, and PECAM-exposing (CD31^+^) EV concentration are not statistically significant independent predictors of hemorrhagic stroke in this model.

**Table 2:** Results of multivariate logistic regression analysis for hemorrhagic stroke classification using age, sex, PECAM-expressing EV concentrations and platelet-derived EV concentrations, based on cutoff values determined with receiver-operating characteristic analysis.

Characteristic	OR (95% CI)	*P*
Age ≥ 67 y	0.52 (0.21-1.31)	.166
Sex	1.60 (0.64-4.15)	.317
CD31^+^ EV concentration ≥ 5.22 × 10^6^/mL	1.77 (0.64-4.85)	.264
CD41^+^ EV concentration ≥ 1.13 × 10^7^/mL	2.83 (1.00-8.09)	**.049**

*P* values shown in bold are statistically significant (*P* < .05).

CD, cluster of differentiation; CI, confidence interval; EV, extracellular vesicle; OR, odds ratio.

**Figure 5:**
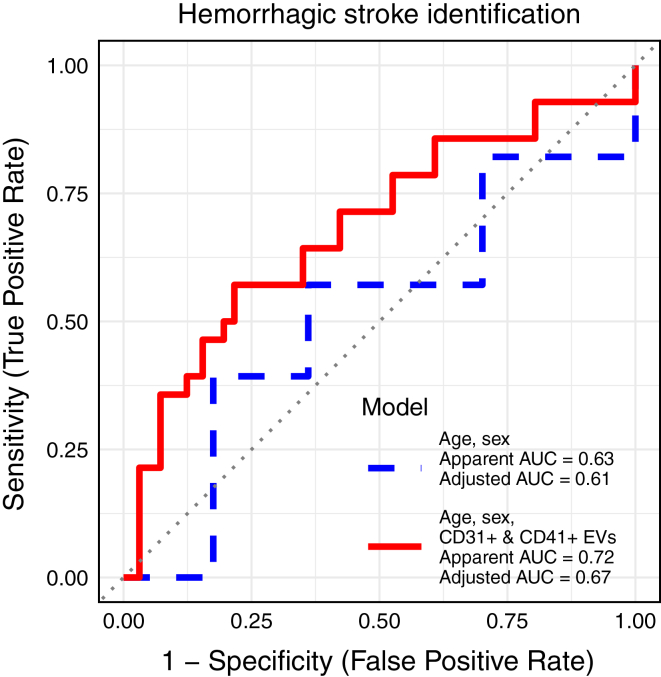
Receiver-operating characteristic (ROC) curves to distinguish between patients with confirmed hemorrhagic stroke and patients with a confirmed different diagnosis. ROC analysis based on age and sex alone (blue dashed line) showed an apparent area under the curve (AUC) of 0.63 and an AUC of 0.61 (95% CI, 0.51-0.71; *P* = .01) after optimism correction using bootstrap validation with 200 resamples. ROC analysis based on age, sex, platelet endothelial cell adhesion molecule–exposing (CD31^+^) extracellular vesicle (EV) concentration, and platelet-derived (CD41^+^) EV concentration (red solid line) showed an apparent AUC of 0.72 and an optimism-adjusted AUC of 0.67 (95% CI, 0.58-0.76; *P* = .01).

The added diagnostic value of the EV concentrations was evaluated by comparing ROC analyses of multiple logistic regression models, including (i) only the baseline parameters age and sex vs (ii) baseline parameters (age and sex) combined with PECAM-exposing (CD31^+^) and platelet-derived (CD41^+^) EV concentrations. Figure 5 shows that adding EV concentrations to the model increased the AUC from 0.63 to 0.72 (*P* = .071, DeLong test), with optimism-adjusted AUCs of 0.61 (95% CI, 0.51-0.71; *P* = .01) and 0.67 (95% CI, 0.58-0.76; *P* = .01), respectively. The borderline significance of the DeLong test may be attributable to the limited sample size of 28 patients with hemorrhagic stroke available for multivariate analysis.

## 4 Discussion

In this study, we revisited 2 independent published clinical studies, AFFECT EV and CINTICS. Measurements in both studies were obtained using sample-specific dilution protocols, a procedure recently shown to introduce systematic bias in EV quantification due to the dilution paradox [5]. The dilution paradox model introduced in our previous work enabled differentiation between reliable and unreliable measurements. After selecting the unbiased data using our analytical model, our revised analysis confirmed that both studies contain biased measurements. In this study, we have reevaluated the diagnostic utility of EVs in the context of myocardial infarction and acute stroke, after addressing the biased EV concentrations that affected previous interpretations of both studies.

### 4.1 Ticagrelor attenuates the release of platelet-derived EVs

In the original AFFECT EV study [6], ticagrelor was shown to significantly attenuate the postinfarction rise of activated platelet-derived (CD61^+^CD62p^+^), fibrinogen^+^, PS-exposing, and leukocyte-derived (CD45^+^) EV concentrations compared with clopidogrel, whereas endothelial-derived (CD31^+^CD146^+^) and erythrocyte-derived (CD235a^+^) EV concentrations remained unchanged. After exclusion of biased data, we found that the magnitude of these differences was partly influenced by overestimated EV concentrations. Application of the dilution paradox model showed that significant differences persisted between ticagrelor and clopidogrel for activated platelet-derived (CD61^+^CD62p^+^) EVs, as the data were 100% reliable. In contrast, any conclusions regarding the clinical utility of fibrinogen^+^ EVs should be disregarded, as 0% of the fibrinogen^+^ EV measurements were reliable. In addition, the originally observed differences in leukocyte-derived (CD45^+^) and PS-exposing EV concentrations between treatment arms were not present upon reanalysis.

While the original AFFECT EV article introduced the concept that ticagrelor’s benefit may extend beyond platelet inhibition [6], our present work did not find evidence potentially reflecting reduced procoagulant and proinflammatory signaling from EVs other than those released by platelets. Together, these results confirm that ticagrelor attenuates the postinfarction release of activated platelet-derived EVs in plasma more effectively than clopidogrel, suggesting that ticagrelor has superior anti-inflammatory properties. However, we found no evidence that recurrent thrombotic events despite antiplatelet therapy or worse clinical outcomes on clopidogrel can be attributed to different fibrinogen^+^, PS-exposing, or leukocyte-derived EV (CD45^+^) concentrations.

### 4.2 EVs as potential biomarkers in stroke subtype identification

In the original CINTICS publication [4], it was reported that plasma concentrations of leukocyte-derived (CD45^+^) EVs and activated platelet-derived (CD62p^+^) EVs differed between patients with ischemic stroke and patients with confirmed different diagnoses, including hemorrhagic stroke. After selecting the unbiased data using the dilution paradox model, our revised analysis revealed that only leukocyte-derived EVs (CD45^+^) retained diagnostic significance for ischemic stroke.

In contrast with the original publication, the difference in activated platelet-derived (CD62p^+^) EV concentration is no longer significant between patients with ischemic stroke and patients with different diagnoses. Ischemic stroke occurs when a thrombus is formed in cerebral vessels, which involves platelet activation [12]. Similar to patients with AMI in the AFFECT EV study, we expected patients with ischemic stroke in the CINTICS cohort to exhibit elevated concentrations of activated platelet-derived (CD62p^+^) EVs. Therefore, the initially reported decrease in activated platelet-derived (CD62p^+^) EV concentrations among patients with ischemic stroke was inconsistent with both the known pathophysiological mechanisms underlying thrombotic events and our prior expectations. After applying the revised model, the differences in activated platelet-derived (CD62p^+^) EV concentrations between patients with and without ischemic stroke were no longer statistically significant, which aligns more closely with our initial hypothesis.

In addition, the present study also compared patients with confirmed hemorrhagic stroke (intracerebral and/or subarachnoid hemorrhage) with those with confirmed other types of strokes. Platelet-derived (CD41^+^) EV concentrations and PECAM-exposing (CD31^+^) EV concentrations showed significant differences between the 2 patient groups. Concentrations of other EV types did not differ significantly between the groups. In multivariate logistic regression analysis, only an elevated concentration of platelet-derived (CD41^+^) EVs was independently associated with higher OR of hemorrhagic stroke. The addition of platelet-derived (CD41^+^) and PECAM-exposing (CD31^+^) EV concentrations to baseline clinical parameters (age and sex) improved the discriminatory power, which suggests an additive, although modest, diagnostic value.

In the original CINTICS study, the comparison between patients with hemorrhagic stroke in general (ie, intracerebral and subarachnoid hemorrhage subgroups together) and patients with a different diagnosis was not made. The comparison between patients with hemorrhagic stroke and other patients with the full dataset yielded a significant difference in the concentrations of platelet-derived (CD41^+^) EVs (*P* = .035) and PECAM-exposing (CD31^+^) EVs (*P* = .042). After model correction, the association between platelet-derived (CD41^+^) and PECAM-exposing (CD31^+^) EVs and hemorrhagic stroke strengthened, due to the exclusion of unreliable data points affected by the dilution paradox (*P* = .006 and *P* = .036 respectively).

The elevated concentrations of platelet-derived (CD41^+^) and PECAM-exposing (CD31^+^) EVs in patients with hemorrhagic stroke are consistent with pathophysiological mechanisms involving acute vascular disruption and platelet/endothelial activation. Hemorrhagic stroke triggers rapid platelet degranulation, activation of endothelial adhesion molecules, and shedding of EVs that carry procoagulant and inflammatory signals [13]. PECAM-exposing (CD31^+^) EVs, derived from activated or damaged endothelium, may reflect vascular wall injury, while increased platelet-derived (CD41^+^) EVs indicate ongoing hemostatic activation at the site of bleeding [14,15].

### 4.3 Model implementation contributes to comparability and reproducibility

Biomarker exploration requires standardized, comparable, and reproducible measurement methods. The recent discovery of the dilution paradox in EV flow cytometry raised concerns about the comparability and reproducibility of previous study results. Indeed, the application of our model to the AFFECT EV and CINTICS studies showed that 46% of the evaluated measurements contained artificially inflated EV concentrations, which leads to neither comparable nor reproducible study conclusions. The exclusion of biased measurements not only strengthened the evidence (platelet-derived [CD41^+^] and PECAM-exposing [CD31^+^] EVs as potential hemorrhagic stroke biomarkers) but also brought the results in closer agreement with prior expectations based on pathophysiological mechanisms. The new model helps to separate true biological signals from technical bias and thus contributes to the improvement of comparability and reproducibility of previously published studies. To overcome the dilution paradox in the future, we recommend following the methodological recommendations given in our previous publication [5].

### 4.4 Limitations

There are several limitations that should be acknowledged. First, the exclusion of data by the model decreased the sample size in all study groups, leading to a possible type II error due to underpowered results. Although the application of the dilution paradox model enabled us to generate hypotheses regarding the clinical utility of EVs after AMI or stroke, conclusions should be validated using an improved measurement procedure that is unaffected by the dilution paradox.

Second, it should be noted that the boundaries defined by the model are estimates rather than true values. The position of the boundaries is determined by the fit of the model and scaled by the standard deviation of the fit. As a result, the exact boundary position can be influenced by all factors that may influence the SD of the measurement, such as flow rate stability or pipetting errors. Therefore, the model-generated boundaries should be interpreted with caution.

## 5 Conclusion

After applying the dilution paradox model, the revised analysis confirmed that 47% (1156/2457) of the evaluated measurements contained artificially inflated EV concentrations as a result of the dilution paradox. Consequently, only a subset of measurements could be reliably assessed for diagnostic value. These revised conclusions emphasize the importance of rigorous analytical reevaluation when exploring EV-based biomarkers.

Reanalysis of the AFFECT EV study confirmed that ticagrelor attenuates the postinfarction release of platelet-derived EVs in plasma more effectively than clopidogrel, although differences in fibrinogen^+^, PS-exposing, or leukocyte-derived (CD45^+^) EV concentrations were no longer statistically significant. Reanalysis of the CINTICS study confirmed that plasma concentrations of leukocyte-derived (CD45^+^) EVs may contribute to the identification of ischemic stroke. Moreover, the present study found that plasma concentrations of platelet-derived (CD41^+^) and PECAM-exposing (CD31^+^) EVs may be useful for the identification of hemorrhagic stroke. EVs remain a promising biomarker class in cardiovascular and cerebrovascular diseases, although clinical translation requires widespread implementation of an improved flow cytometry measurement procedure.

More importantly, the dilution paradox model effectively identifies originally biased data and provides corrected study data with improved accuracy and reproducibility. We recommend that other researchers adopt this correction method when analyzing EV flow cytometry data obtained with custom sample dilutions. Incorporating this approach to reanalyze flow cytometry studies will help ensure data comparability across studies, minimize measurement bias, and improve the reliability of quantitative results.
