# Supplementary material for: Resolving the dilution paradox to improve the interpretation of extracellular vesicle biomarker studies
Source: Res Pract Thromb Haemost. 2026 May 8;10(4):106636. doi: 10.1016/j.rpth.2026.106636 (PMC13325907; doi:10.1016/j.rpth.2026.106636)
Supplement: Supplementary Data 1 [file mmc1.docx]

| **Supplemental Table 1:** Comparison of baseline characteristics of included patients with confirmed ischemic stroke and patients with a confirmed different diagnosis. Normally distributed data is represented as mean ± standard deviation and is compared using Welch’s t-tests. Non-normally distributed data is represented as median (95% confidence interval) and is compared using Mann-Whitney U tests. Categorical data is represented as percentage of patients and is compared using Fisher’s Exact test. | | | | | |
| --- | --- | --- | --- | --- | --- |
|  | Total population  (n=115) | Ischemic stroke  (n=55) | Other diagnoses  (n=60) | Data complete | P-value |
| **Baseline characteristics** | | | | | |
| Age | 70 (60-78) | 71 (64-79) | 67.5 (54.5-76) | 100% | 0.06 |
| Sex (%women) | 53.9% (62) | 52.7% (29) | 55.0% (33) | 100% | 0.85 |
| BMI | 26.5 ± 5.0 | 27.4 ± 6.7 | 25.8 ± 3.3 | 38% | 0.33 |
| SBP | 155.5 ± 26.9 | 158.4 ± 23.5 | 152.8 ± 29.6 | 100% | 0.26 |
| DBP | 87.9 ± 17.7 | 91.1 ± 18.8 | 85.0 ± 16.2 | 100% | 0.06 |
| **Co-morbidities, % (n)** | | | | | |
| Prior IS / TIA | 28.7% (29) | 26.4% (14) | 31.25% (15) | 88% | 0.66 |
| Atrial fibrillation | 19.8% (20) | 22.6% (12) | 16.7% (8) | 88% | 0.62 |
| Diabetes Mellitus | 17.8 (18) | 17.0% (9) | 18.8 (9) | 88% | 1.00 |
| Hypercholesterolemia | 16.8% (17) | 17.0% (9) | 16.7% (8) | 88% | 1.00 |
| Hypertension | 60.4% (40) | 62.3% (33) | 58.3% (28) | 88% | 0.84 |
| Coronary artery disease | 15.8% (16) | 18.9% (10) | 12.5% (6) | 88% | 0.43 |
| Past / current smoker | 43.6% (44) | 49.1% (26) | 37.5% (18) | 88% | 0.32 |
| Active cancer | 12.5% (10) | 9.8% (4) | 15.4% (6) | 70% | 0.51 |
| **Pharmacotherapy, % (n)** | | | | | |
| Antiplatelet therapy | 69.6% (39) | 69.0% (20) | 70.4% (19) | 49% | 1.00 |
| Vitamin K antagonist | 8.9% (5) | 6.9% (2) | 11.1% (3) | 49% | 0.66 |
| DOAC | 21.4% (12) | 20.7% (6) | 22.2% (6) | 49% | 1.00 |
| Antihypertensive drugs | 51.3% (58) | 56.4% (31) | 46.6% (27) | 98% | 0.35 |
| Statin | 38.4% (43) | 43.4% (23) | 33.9% (20) | 97% | 0.34 |
| **Laboratory data** | | | | | |
| Platelets (10^3^ μl^-1^) | 255 (219-290) | 250.5 (225-282) | 259 (211-306.5) | 99% | 0.44 |
| Leukocytes (10^3^ μl^-1^) | 8.1 (6.8-10.7) | 8 (6.4-9.5) | 8.3 (6.9-10.9) | 98% | 0.22 |
| CRP (mg L^-1^) | 2.6 (0.7-6.65) | 2.4 (0.7-8.2) | 2.7 (0.6-6.6) | 97% | 0.65 |
| Glucose (mg L^-1^) | 6.95 (5.95-8.6) | 7 (6.1-7.9) | 6.85 (5.9-10.4) | 97% | 0.47 |
| **Diagnosis, treatment, outcome** | | | | | |
| NIHSS (points) | 4 (1-7) | 5 (3-9.5) | 1 (0-2) | 48% | **<0.0001** |
| Occlusion on CT | 40% (36) | 70% (35) | 2.5 (1) | 78% | **<0.0001** |
| Reperfusion therapy | 25.2% (29) | 50.9% (28) | 1.7% (1) | 100% | **<0.0001** |
| IVT | 62.1% (18) | 60.7% (17) | 100% (1) |  |  |
| IAT | 51.7 (15) | 53.6% (15) | 0% (0) |  |  |
| Mortality 7 days, % (n) | 10.5% (11) | 8.5% (4) | 12.1% (7) | 91% | 0.751 |
| BMI = body mass index; CRP = C-reactive protein; CT = computed tomography; DBP = diastolic blood pressure; DOAC = direct oral anticoagulant; IAT = intra-arterial thrombectomy; IS = ischemic stroke; IVT = intravenous thrombolysis; NIHSS = national institutes of health stroke scale; SBP = systolic blood pressure; TIA = transient ischemic attack. | | | | | |

| **Supplemental table 2:** Comparison of baseline characteristics of all patients who were diagnosed with hemorrhagic stroke and patients who received a different diagnosis. Normally distributed data is represented as mean ± standard deviation and is compared using Welch’s t-tests. Non-normally distributed data is represented as median (95% confidence interval) and is compared using Mann-Whitney U tests. Categorical data is represented as percentage of patients and is compared using Fisher’s Exact test. | | | | | |
| --- | --- | --- | --- | --- | --- |
|  | Total population  (n=159) | Hemorrhagic stroke  (n=34) | Other diagnoses  (n=125) | Data complete | P-value |
| **Baseline characteristics** | | | | | |
| Age | 68 (57-76) | 66 (53.5-76) | 68 (58-78) | 100% | 0.48 |
| Sex (%female) | 50.9% (81) | 64.7% (22) | 47.2% (59) | 100% | 0.08 |
| BMI | 26.5 (23.3-29.7) | 24.6 (23.3-29.1) | 26.7 (23.3-30.2) | 40% | 0.47 |
| SBP | 139.5 ± 27.0 | 163.3 ± 34.3 | 155.1 ± 25.0 | 93% | 0.25 |
| DBP | 89.4 ± 17.3 | 90.3 ± 18.2 | 89.2 ± 17.2 | 93% | 0.78 |
| **Co-morbidities, % (n)** | | | | | |
| Prior stroke | 32.1% (44) | 25.9% (7) | 33.6% (37) | 86% | 0.50 |
| Prior IS / TIA | 28.5% (39) | 18.5% (5) | 30.9% (34) | 86% | 0.24 |
| Prior HS | 3.6% (5) | 7.4% (2) | 2.7% (3) | 86% | 0.26 |
| Prior SAH | 2.9% (4) | 7.4% (2) | 1.8% (2) | 86% | 0.17 |
| Atrial fibrillation | 16.1% (22) | 11.1% (3) | 17.3% (19) | 86% | 0.57 |
| Diabetes Mellitus | 21.9% (30) | 18.5% (5) | 22.7% (25) | 86% | 0.80 |
| Hypercholesterolemia | 16.8% (23) | 14.8% (4) | 17.3% (19) | 86% | 1.00 |
| Hypertension | 59.1% (81) | 55.6% (15) | 60.0% (66) | 86% | 0.67 |
| Coronary artery disease | 13.9% (19) | 14.8% (4) | 13.6% (15) | 86% | 1.00 |
| Past / current smoker | 40.9% (56) | 25.9% (7) | 44.5% (49) | 86% | 0.09 |
| Active cancer | 12.8% (15) | 21.7% (5) | 10.6% (10) | 74% | 0.17 |
| **Pharmacotherapy, % (n)** | | | | | |
| Antiplatelet therapy | 69.9% (51) | 45.5% (5) | 74.2% (46) | 46% | 0.08 |
| Vitamin K antagonist | 9.6% (7) | 27.3% (3) | 6.5% (4) | 46% | 0.06 |
| DOAC | 20.5% (15) | 27.3% (3) | 19.3% (12) | 46% | 0.69 |
| Antihypertensive drugs | 49.4% (77) | 42.4% (14) | 51.2% (63) | 98% | 0.43 |
| Statin | 33.8% (52) | 18.8% (6) | 37.7% (46) | 97% | 0.06 |
| **Laboratory data** | | | | | |
| Platelets (10^3^ μl^-1^) | 253 (212-288) | 277 (204.75-331.25) | 252 (212-281) | 97% | 0.18 |
| Leukocytes (10^3^ μl^-1^) | 8.2 (6.7-10.7) | 10.6 (7.6-13.4) | 7.9 (6.6-9.7) | 97% | **<0.001** |
| CRP (mg L^-1^) | 2.5 (0.8-5.9) | 4.0 (1.0-7.5) | 2.0 (0.7-5.7) | 96% | 0.17 |
| Glucose (mg L^-1^) | 7.1 (6.0-9.2) | 9.1 (6.4-11.5) | 6.7 (5.9-8.8) | 96% | **0.02** |
| **Diagnosis and outcome** | | | | | |
| NIHSS (points) | 4 (1-7) | 4 (0-5) | 3.5 (1.0-7.3) | 43% | 0.63 |
| Mortality 7 days, %(n) | 11.5% (17) | 36.4% (12) | 4.3% (5) | 93% | **<0.0001** |
| BMI = body mass index; CRP = C-reactive protein; CT = computed tomography; DBP = diastolic blood pressure; DOAC = direct oral anticoagulant; IS = ischemic stroke; NIHSS = national institutes of health stroke scale; SBP = systolic blood pressure; TIA = transient ischemic attack. | | | | | |

###

| **Supplemental table 3:** Comparison of baseline characteristics of included patients after data correction, who were diagnosed with hemorrhagic stroke and patients who received a different diagnosis. Normally distributed data is represented as mean ± standard deviation and is compared using Welch’s t-tests. Non-normally distributed data is represented as median (95% confidence interval) and is compared using Mann-Whitney U tests. Categorical data is represented as percentage of patients and is compared using Fisher’s Exact test. | | | | | |
| --- | --- | --- | --- | --- | --- |
|  | Total population  (n=121) | Hemorrhagic stroke  (n=26) | Other diagnoses  (n=95) | Data complete | P-value |
| **Baseline characteristics** | | | | | |
| Age | 69 (58-78) | 66 (54-75) | 70 (61-79) | 100% | 0.15 |
| Sex (%female) | 55% (67) | 65% (17) | 53% (50) | 100% | 0.27 |
| BMI | 25.7 (23.2-29.2) | 26.5 (23.5-29.1) | 25.1 (22.4-29.4) | 40% | 0.82 |
| SBP | 155.0 ± 27.0 | 155.8 ± 33.1 | 154.8 ± 25.6 | 93% | 0.90 |
| DBP | 86 (75-99.5) | 86.5 (75.5-99.5) | 85.5 (75-99.5) | 93% | 0.96 |
| **Co-morbidities, % (n)** | | | | | |
| Prior stroke | 33.6% (36) | 30.0% (6) | 34.5% (30) | 88% | 0.80 |
| Prior IS / TIA | 29.0% (31) | 20.0% (4) | 31.0% (27) | 88% | 0.42 |
| Prior HS | 4.7% (5) | 10.0% (2) | 3.4% (3) | 88% | 0.23 |
| Prior SAH | 3.7% (4) | 10.0% (2) | 2.3% (2) | 88% | 0.16 |
| Atrial fibrillation | 81.3% (87) | 90.0% (18) | 79.3% (69) | 88% | 0.35 |
| Diabetes Mellitus | 17.8% (19) | 10% (2) | 19.5% (17) | 88% | 0.52 |
| Hypercholesterolemia | 17.8% (19) | 20.0% (4) | 17.2% (15) | 88% | 0.75 |
| Hypertension | 57.9% (62) | 60.0% (12) | 57.5% (50) | 88% | 1.00 |
| Coronary artery disease | 15.0% (16) | 15.0% (3) | 15.0% (13) | 88% | 1.00 |
| Past / current smoker | 43.9% (47) | 30.0% (6) | 47.1% (41) | 88% | 0.21 |
| Active cancer | 14.1% (12) | 4.7% (4) | 9.4% (8) | 70% | 0.25 |
| **Pharmacotherapy, % (n)** | | | | | |
| Antiplatelet therapy | 27.1% (16) | 44.4% (4) | 24.0% (12) | 49% | 0.24 |
| Vitamin K antagonist | 8.5% (5) | 22.2% (2) | 6.0% (3) | 49% | 0.16 |
| DOAC | 20.3% (12) | 22.2% (2) | 20.0% (10) | 49% | 1.00 |
| Antihypertensive drugs | 50.4% (60) | 38.5% (10) | 53.8% (50) | 98% | 0.19 |
| Statin | 38.1% (45) | 23.1% (6) | 42.4% (39) | 98% | 0.11 |
| **Laboratory data** | | | | | |
| Platelets (10^3^ μl^-1^) | 255 (210-291) | 285 (221-310) | 252.5 (210-282) | 98% | 0.18 |
| Leukocytes (10^3^ μl^-1^) | 8.05 (6.8-10.7) | 10.7 (8.0-13.1) | 7.9 (6.7-9.5) | 98% | **<0.001** |
| CRP (mg L^-1^) | 2.5 (0.7-6.25) | 4.2 (0.8-6.7) | 2.0 (0.6-5.9) | 96% | 0.27 |
| Glucose (mg L^-1^) | 7.05 (5.9-8.8) | 8.95 (6.35-11.2) | 6.65 (5.8-8.1) | 98% | **0.02** |
| **Diagnosis and outcome** | | | | | |
| NIHSS (points) | 4 (1-7) | 4.5 (4-5) | 4 (1-7) | 45 | 0.77 |
| Mortality 7 days, %(n) | 11.7% (13) | 36.0% (9) | 4.7% (4) | 92 | **<0.001** |
| BMI = body mass index; CRP = C-reactive protein; CT = computed tomography; DBP = diastolic blood pressure; DOAC = direct oral anticoagulant; IS = ischemic stroke; NIHSS = national institutes of health stroke scale; SBP = systolic blood pressure; TIA = transient ischemic attack. | | | | | |

###

**Supplemental table 4:** Univariate receiver operator characteristics analysis to determine optimal cut-off values for the diagnosis of ischemic stroke.

|  | **AUC (95% CI)** | **Adjusted AUC (95% CI)** | **p-value** | **Cut-off** | **Sensitivity** | **Specificity** | **PPV** | **NPV** |
| --- | --- | --- | --- | --- | --- | --- | --- | --- |
| Age (years) | 0.60 (0.50-0.70) | 0.60 (0.50-0.70) | **0.03** | 58 years | 89% | 32% | 58% | 61% |
| DBP (mmHg) | 0.60 (0.50-0.71) | 0.61 (0.51-0.72) | 0.07 | 81 mmHg | 71% | 48% | 54% | 76% |
| CD45+ EVs (mL^-1^) | 0.60 (0.49-0.70) | 0.59 (0.50-0.68) | 0.07 | 7.74∙10^6^ mL^-1^ | 64% | 57% | 56% | 64% |

AUC = area under the curve; CD = cluster of differentiation; CI = confidence interval; EV = extracellular vesicle; NPV = negative predictive value; PPV = positive predictive value.

**Supplemental table 5:** Univariate receiver operator characteristics analysis to determine optimal cut-off values for the diagnosis of hemorrhagic stroke.

|  | **AUC (95% CI)** | **Adjusted AUC (95% CI)** | **p-value** | **Cut-off** | **Sensitivity** | **Specificity** | **PPV** | **NPV** |
| --- | --- | --- | --- | --- | --- | --- | --- | --- |
| Age (years) | 0.59 (0.47-0.72) | 0.60 (0.48-0.74) | 0.138 | 67 years | 57% | 64% | 34% | 86% |
| Gender | 0.56 (0.46-0.67) | 0.54 (0.46-0.62) | 0.236 | - | 64% | 48% | 31% | 84% |
| CD31+ EVs (mL^-1^) | 0.63 (0.50-0.75) | 0.61 (0.50-0.74) | 0.213 | 5.22∙10^6^ mL^-1^ | 61% | 65% | 31% | 84% |
| CD41+ EVs (mL^-1^) | 0.67 (0.54-0.79) | 0.67 (0.56-0.85) | 0.115 | 1.13∙10^7^ mL^-1^ | 50% | 81% | 33% | 85% |

AUC = area under the curve; CD = cluster of differentiation; CI = confidence interval; EV = extracellular vesicle; NPV = negative predictive value; PPV = positive predictive value.


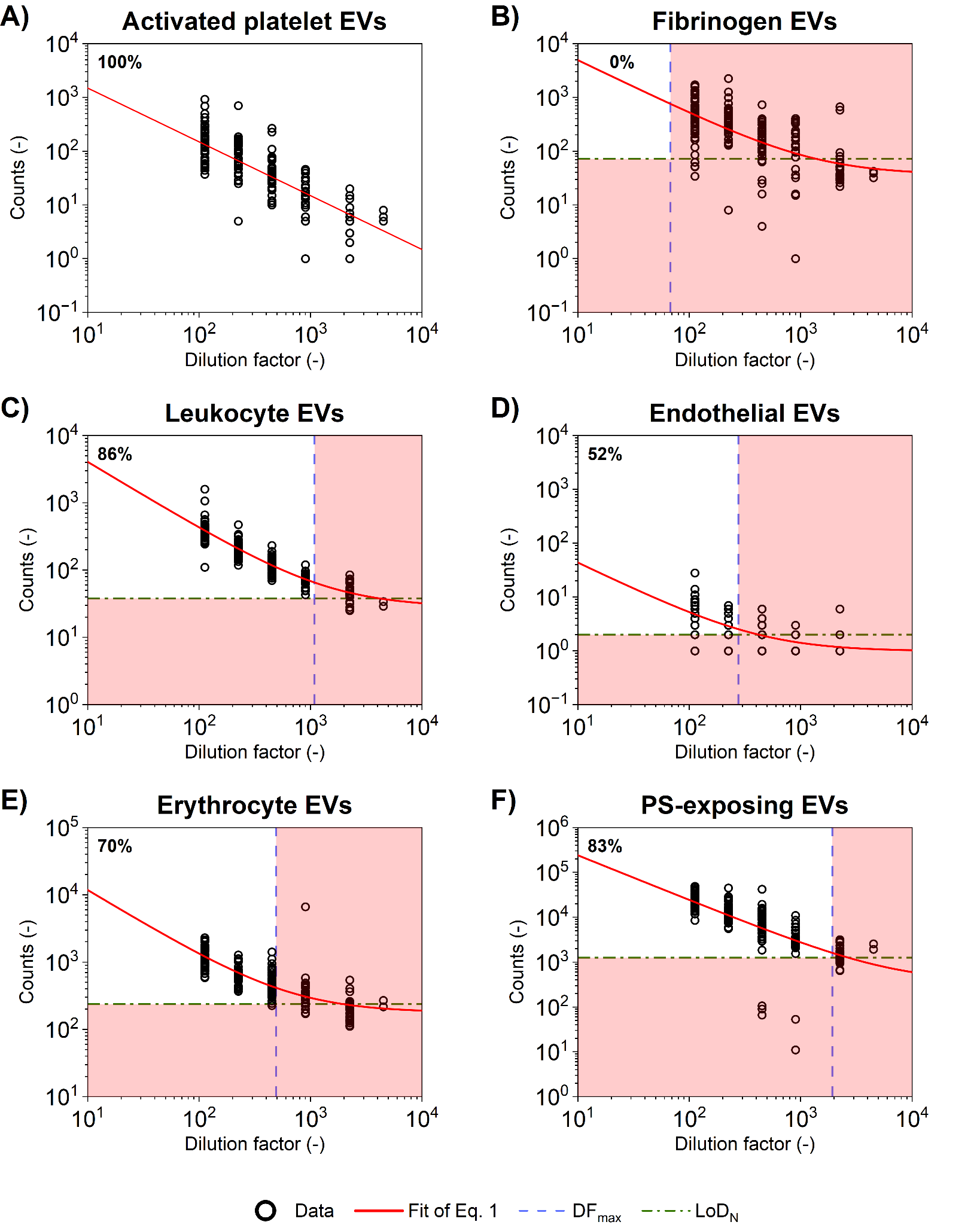


**Supplemental figure 1:** Application of model to data from the AFFECT EV study. Data represent measured number of events that exceeded the side scattering cross section threshold of 10 nm^2^, have a diameter between 100 and 1000 nm and are positive for the indicated marker versus dilution factor. The solid red line represents the fit of Equation 1 to the data. The vertical dashed line represents $DF_{max}$ and the horizontal dashed-dotted line represents $LoD_{N}$. All data within the shaded area are considered unreliable. The percentages in the top left corners indicate the percentage of reliable measurements for that marker. (A) CD61-APC & CD62p-PE: $LoD_{N}$ = 4 counts, $DF_{max}$ = 4.12·10^3^, (B) Fibrinogen-FITC: $LoD_{N}$ = 72 counts, $DF_{max}$ = 6.78·10^1^, (C) CD45-APC: $LoD_{N}$ = 38 counts, $DF_{max}$ = 1.09·10^3^, (D) CD31-APC & CD146-PE: $LoD_{N}$ = 2 counts, $DF_{max}$ = 2.76·10^2^, (E) CD235a-PE: $LoD_{N}$ = 238 counts, $DF_{max}$ = 4.92·10^2^, (F) Lactadherin-FITC: $LoD_{N}$ = 1251 counts, $DF_{max}$ = 1.92·10^3^. APC = allophycocyanin; CD: cluster of differentiation; $DF_{max}$ = maximum dilution factor; FITC = fluorescein isothiocyanate; $LoD_{N}$ = limit of detection in terms of counts; PE = phycoerythrin; PS = phosphatidylserine.

###
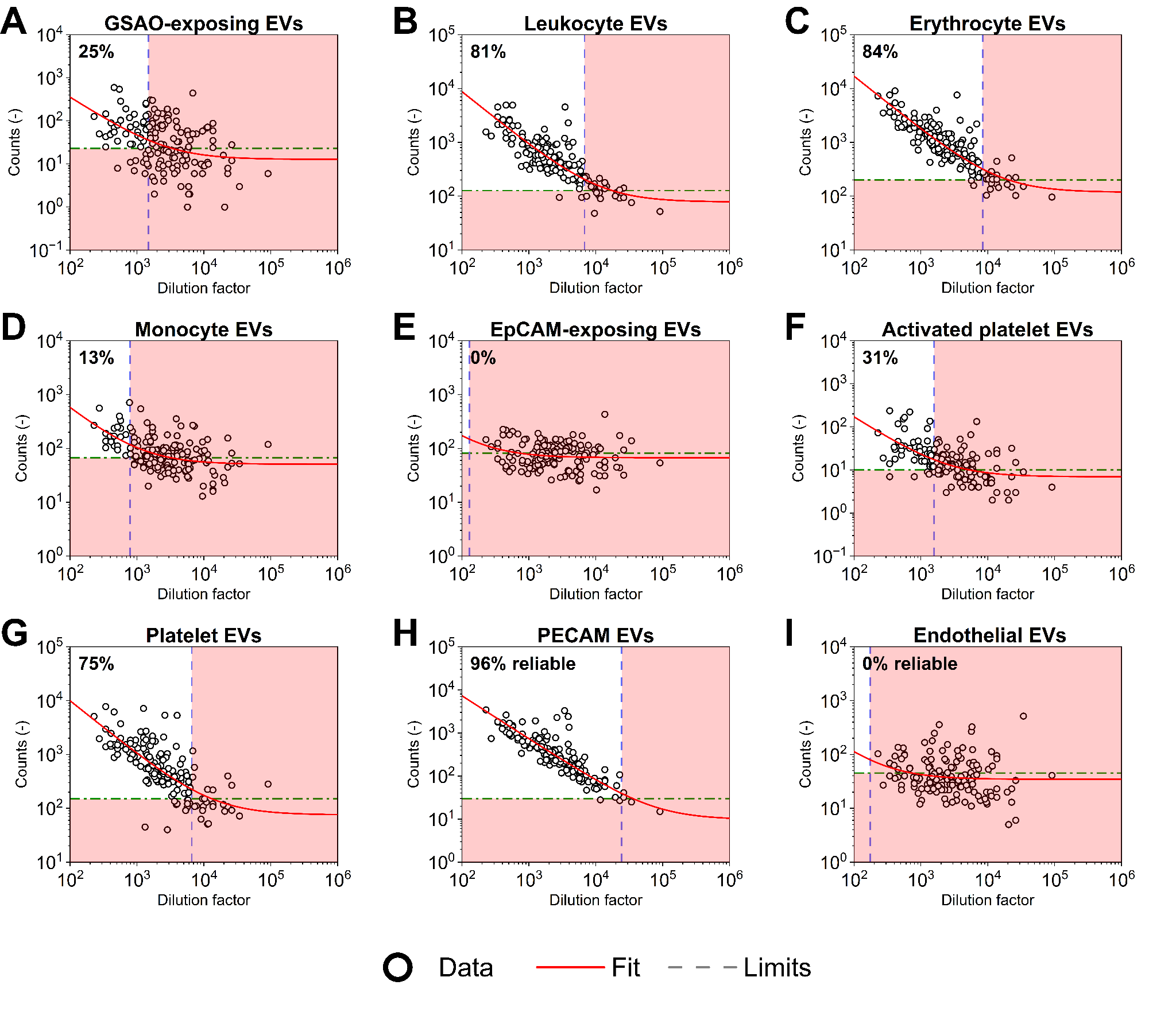


**Supplemental figure 2:** Application of model to CINTICS data. Data represent measured number of events that exceeded the side scattering cross section threshold of 2 nm^2^, have a diameter between 100 and 1000 nm and are positive for the indicated marker versus dilution factor. The solid red line represents the fit of Equation 1 to the data. The vertical dashed line represents $DF_{max}$ and the horizontal dashed-dotted line represents $LoD_{N}$. All data within the shaded area are considered unreliable. The percentages in the top left corners indicate the percentage of reliable measurements for that marker. (A) GSAO-AF647: $LoD_{N}$ = 23 counts, $DF_{max}$ = 1.49·10^3^, (B) CD45-APC: $LoD_{N}$ = 127 counts, $DF_{max}$ = 6.79·10^3^, (C) CD235a-PE: $LoD_{N}$ = 201 counts, $DF_{max}$ = 8.41·10^3^, (D) CD14-PacificBlue: $LoD_{N}$ = 67 counts, $DF_{max}$ = 7.88·10^2^, (E) CD326-APC: $LoD_{N}$ = 82 counts, $DF_{max}$ = 1.28·10^2^, (F) CD62p-PE: $LoD_{N}$ = 10 counts, $DF_{max}$ = 1.58·10^3^, (G) CD41-PacificBlue: $LoD_{N}$ = 150 counts, $DF_{max}$ = 6.59·10^3^, (H) CD31-APC: $LoD_{N}$ = 30 counts, $DF_{max}$ = 2.43·10^4^, (I) CD146-PE: $LoD_{N}$ = 45 counts, $DF_{max}$ = 1.74·10^2^. APC = allophycocyanin; CD: cluster of differentiation; $DF_{max}$ = maximum dilution factor; EpCAM = Epithelial Cell Adhesion Molecule; GSAO = 4-[N-(S-glutathionylacetyl)amino]phenylarsonous acid; $LoD_{N}$ = limit of detection in terms of counts; PE = phycoerythrin; PECAM = Platelet Endothelial Cell Adhesion Molecule.
